# Osteosarcoma of the Distal Femur Presenting as Giant Cell Tumor: A Case Report

**DOI:** 10.7759/cureus.33173

**Published:** 2022-12-31

**Authors:** Salahuddin Ahmed, Sanjay V Deshpande, Kuldeep Chhatbar

**Affiliations:** 1 Department of Orthopaedics, Jawaharlal Nehru Medical College, Datta Meghe Institute of Higher Education & Research, Wardha, IND

**Keywords:** denosumab, aneurysmal bone cyst, osteogenic sarcoma, malignant transformation, giant cell tumour

## Abstract

The most frequent benign bone tumor, known as a giant cell tumor (GCT), typically develops in the second and third decades of life. GCTs of the bone that have already been diagnosed and have already undergone treatment with denosumab therapy, curettage or excision, or radiotherapy frequently develop malignant transformation. A very uncommon occurrence involves a GCT of the bone that has always been malignant. Here, we describe the case of a 25-year-old man with a large cell tumor of the distal femur discovered after six months of symptom onset. The MRI suggested an aneurysmal bone cyst or subsequent modifications of an aneurysmal bone cyst in a GCT. A biopsy was performed, and the results pointed to a benign GCT of the bone. There were no pleomorphic or hyperchromatic lesions, unusual mitoses, or cellular atypia. The patient was treated with tumor removal and internal fixation using plate osteosynthesis two weeks later. The samples were sent for histopathology. The report was suggestive of osteogenic sarcoma or malignant transformation of the giant cell. This could happen due to the possibility of a biopsy sample being taken from an area not representative of the tumor site, which is not uncommon since the osteosarcoma also contains areas of conventional GCT. Thus, osteosarcoma usually mimics conventional GCT of the bone.

## Introduction

The most frequent benign bone tumor, known as a giant cell tumor (GCT), usually appears in the age group of 20-30 years. It frequently occurs in the epiphysis-metaphyseal region of long bones. The distal femur and proximal tibia are the most commonly encountered sites, followed by the distal radius [1]. Despite being benign, the lesion is aggressive locally and has the potential to invade nearby soft tissues. Histologically, the mononuclear cells that make up the GCT are dotted with multinucleated large cells. The biological behavior of the tumor and its histological appearance may not necessarily correlate. Surgery has generally been considered the best course of treatment. The tumor can be removed either through en bloc excision or intralesional curettage. The goal of treatment is to maintain function while reducing the likelihood of recurrence [2]. The likelihood of a recurrence is influenced by the degree of curettage and tumor features, including tumor site and size. Receptor-activated nuclear factor Kappa (RANK) is expressed by the multinucleated GCT. The mesenchymal cancer cells produce RANK ligands (RANKL). The interaction of these RANK-RANKL triggers the differentiation of neoplastic cells in GCT, which gives the tumor its aggressive nature and destroys the bone [3,4]. Recently, denosumab therapy has been suggested to treat bone GCT. A humanized monoclonal antibody called denosumab blocks the RANK-RANKL connection, preventing tumor differentiation. Most of these malignant changes happened when tumors recurred following radiation therapy, denosumab therapy, or surgical curettage [5,6].

Reports of primary bone GCTs that have undergone high-grade malignant transformation without having previously had surgery, radiation therapy, or denosumab therapy are rare. Some publications have referred to this event as bone GCT dedifferentiation [3,7]. However, there is very little evidence to support this theory. A more common theory available in the literature is that malignant GCT often contains areas of conventional GCT. The initial biopsy could have been taken from these sites, and the diagnosis could have been missed. In this case report, we present a patient who visited a tertiary care hospital with complaints of knee pain and swelling for one year. The X-ray of the distal femur showed a lytic lesion. The MRI suggested an aneurysmal bone cyst or subsequent modifications of an aneurysmal bone cyst in a GCT. A benign GCT of the bone without sarcomatous characteristics was discovered during an incisional biopsy. After being removed two weeks later, the tumor revealed sections of high-grade osteosarcoma and benign GCT.

## Case presentation

**Figure 1:**
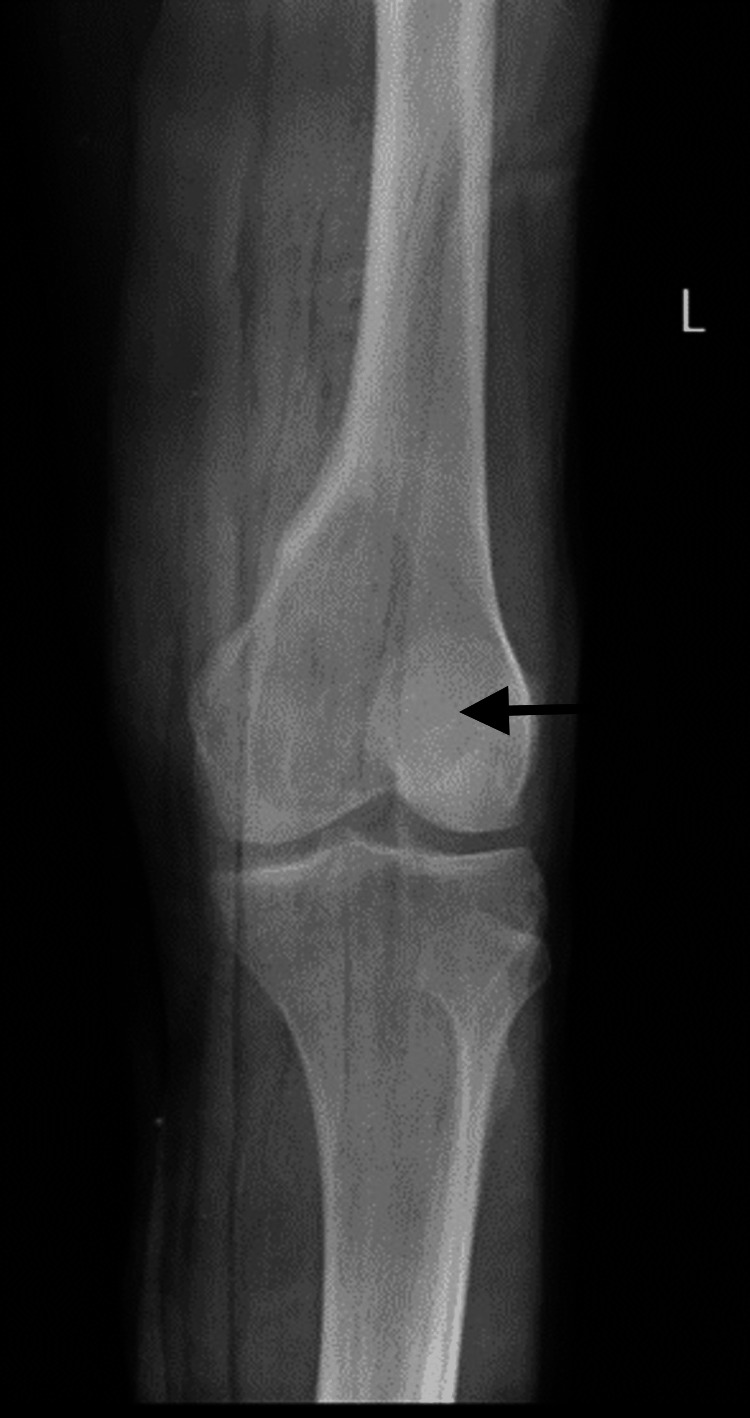
Plain radiograph of the knee, anteroposterior view shows lytic lesion over the lateral femoral condyle

**Figure 2:**
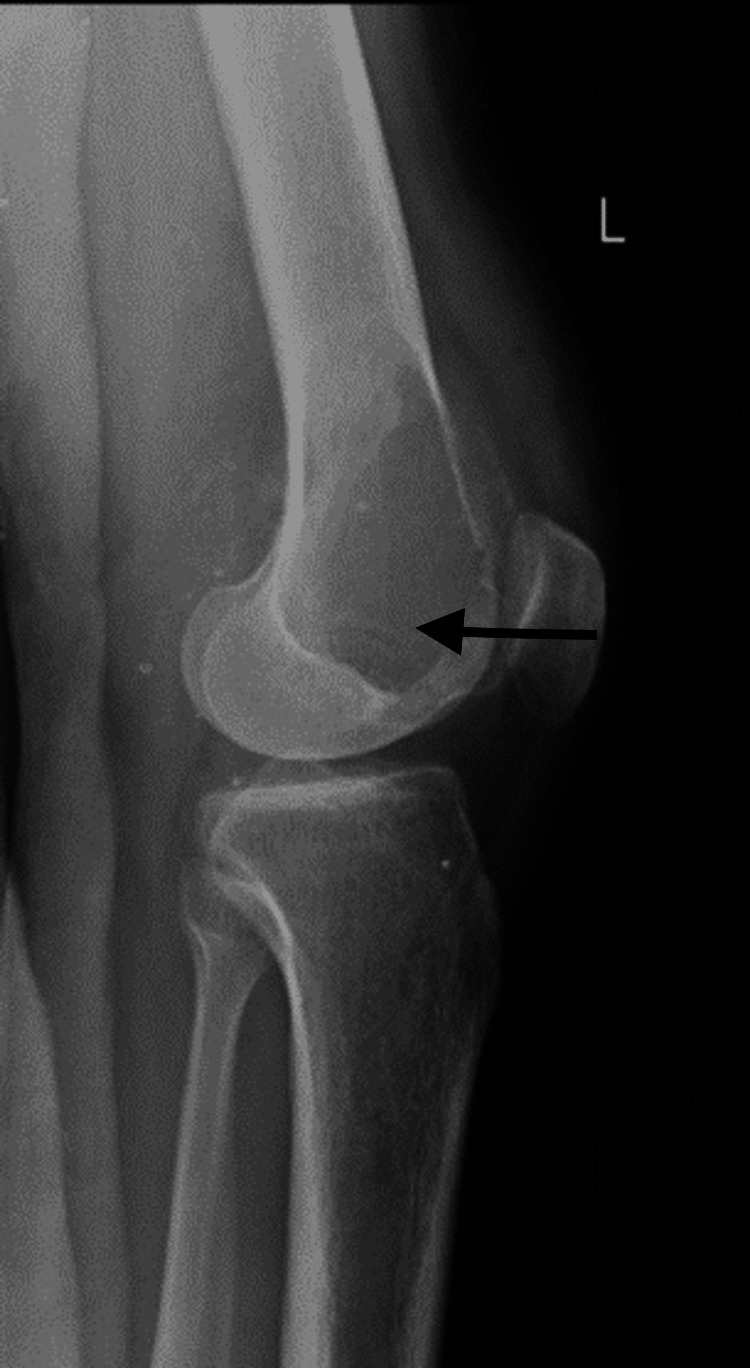
Plain radiograph of the knee, lateral view shows lytic lesion over the lateral femoral condyle

A 25-year-old male patient arrived at the Acharya Vinoba Bhave Rural Hospital (AVBRH) complaining of discomfort and swelling in his left knee for one year, which had worsened during the previous month. A widespread, poorly defined enlargement measuring 6 x 4 cm was seen during a clinical examination over the left lateral femoral condyle. Anteroposterior and lateral knee views on a plain radiograph revealed a lytic lesion in the lateral condyle of the femur (Figures 1, 2).

**Figure 3:**
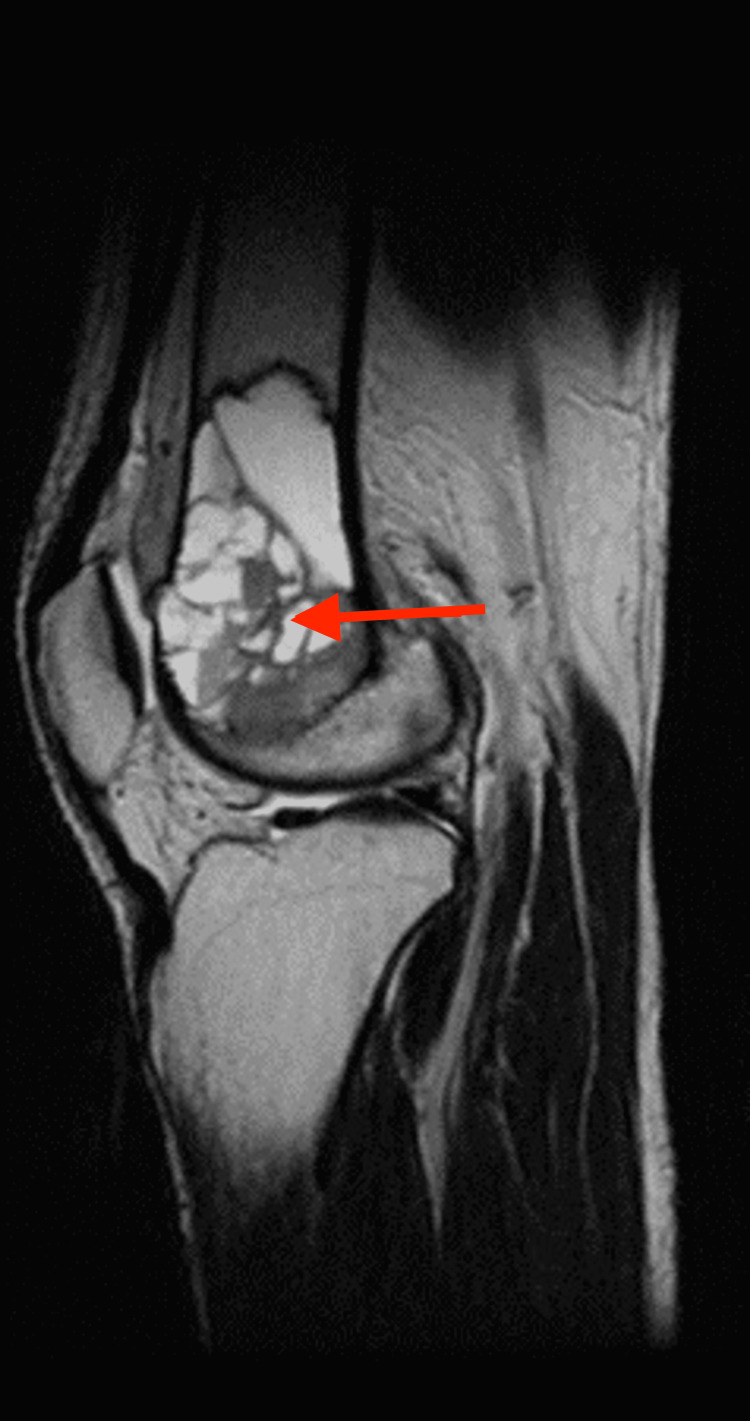
MRI of the left knee showing well-defined multiloculated expansile altered signal intensity mass lesion at the distal end of femur appearing hyperintense on T2WI, variable intensity on T1WI, showing fluid-fluid levels within the locules with areas of blooming on T2 and hyperintensities in T1WI suggestive of hemorrhagic components

**Figure 4:**
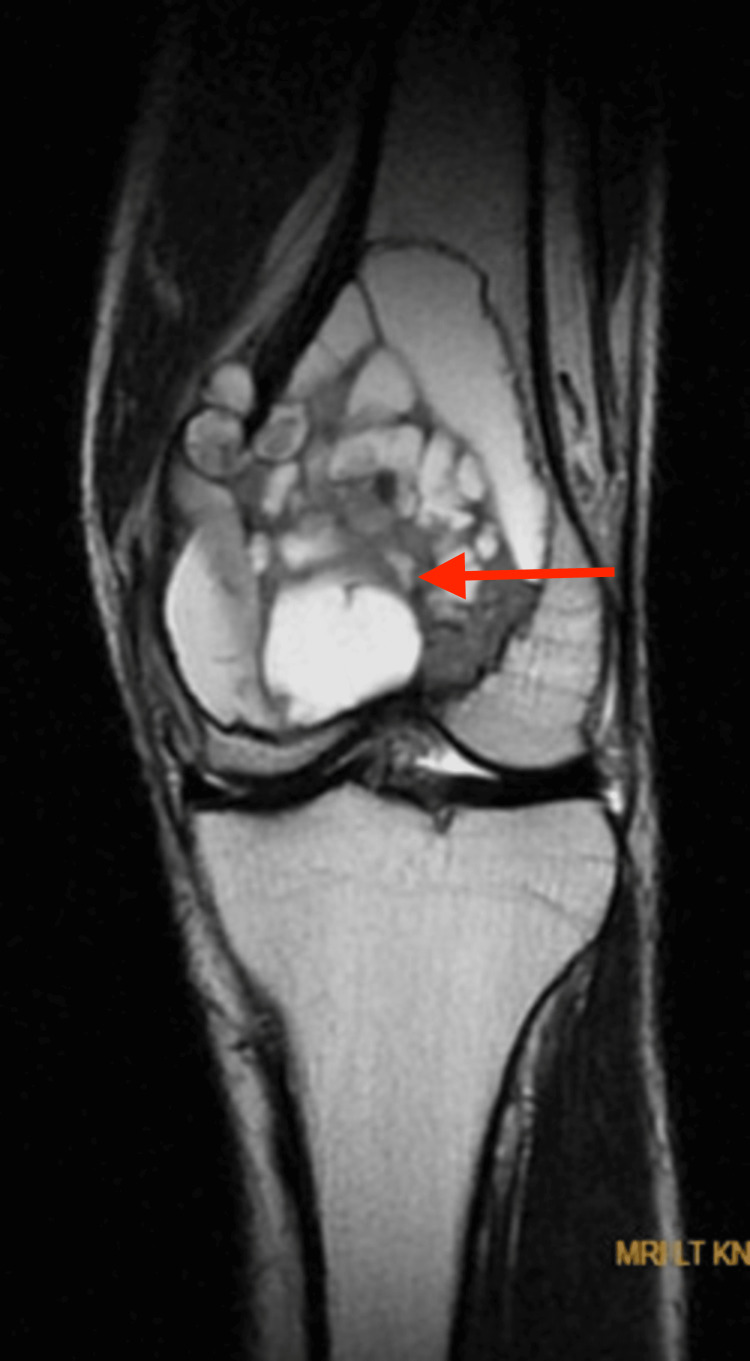
MRI of the left knee showing well-defined multiloculated expansile altered signal intensity mass lesion at the distal end of femur appearing hyperintense on T2WI, variable intensity on T1WI, showing fluid-fluid levels within the locules with areas of blooming on T2 and hyperintensities in T1WI suggestive of hemorrhagic components

The patient was admitted with a presumptive diagnosis of either a GCT or an aneurysmal bone cyst. An MRI of the left knee was performed, and the results suggested either an aneurysmal bone cyst or subsequent alterations of an aneurysmal bone cyst in a GCT. The lesion was highly defined, expansile, multilobulated, and had multiple fluid levels (Figures 3, 4).

**Figure 5:**
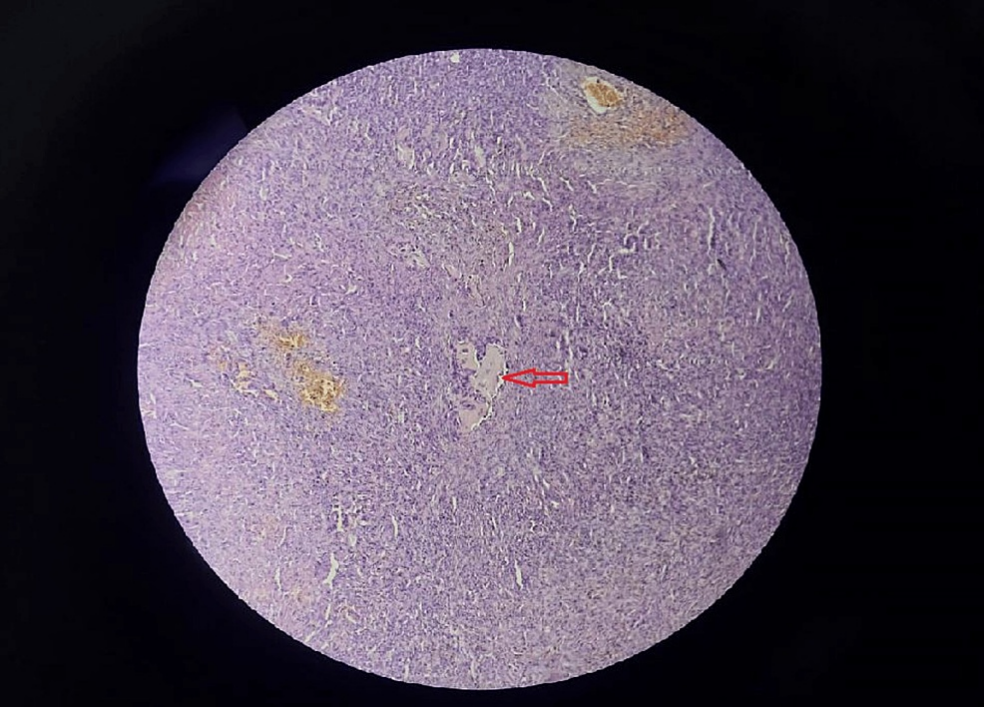
Biopsy material demonstrated features of an aneurysmal bone cyst with bland stromal cells with osteoclast-like giant cells (H&E, x125)

**Figure 6:**
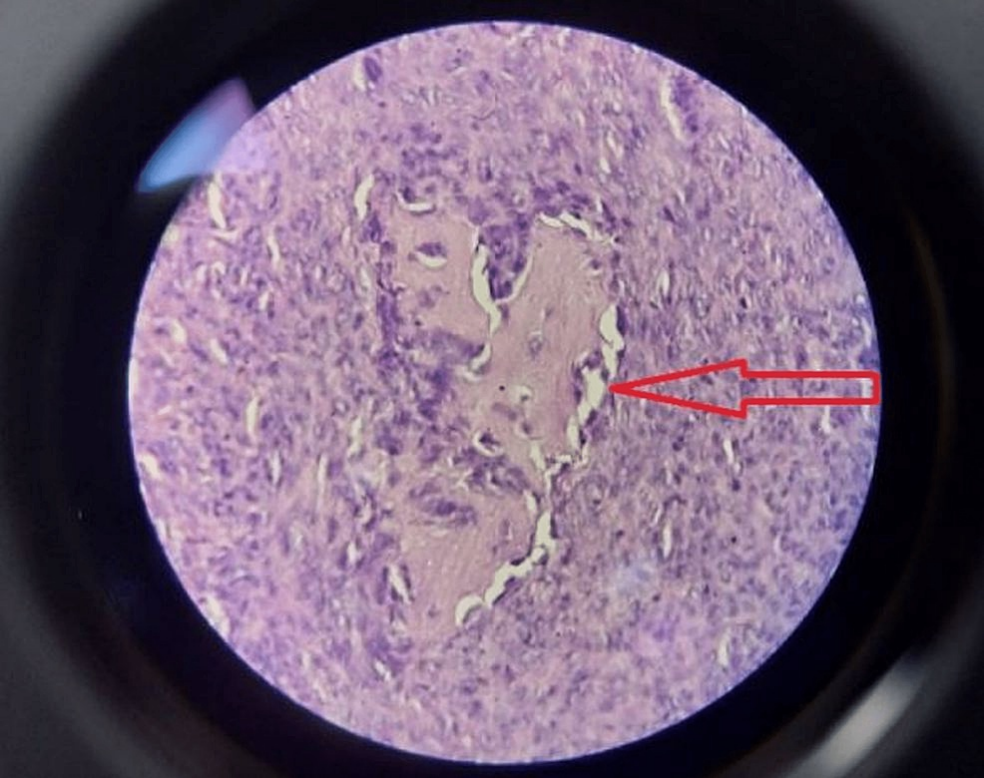
High power examination shows features of an aneurysmal bone cyst with bland stromal cells with osteoclast-like giant cells (H&E, x250)

On June 23, 2022, an incisional biopsy under spinal anesthesia was carried out. Slides from the biopsy revealed sections of aneurysmal bone cysts (ABC) with a lot of hemosiderin pigment, bland stromal cells, and osteoclast-like cells (Figures 5, 6). All of these characteristics pointed to the aneurysmal bone cyst. There were no pleomorphic or hyperchromatic lesions, unusual mitoses, or cellular atypia.

**Figure 7:**
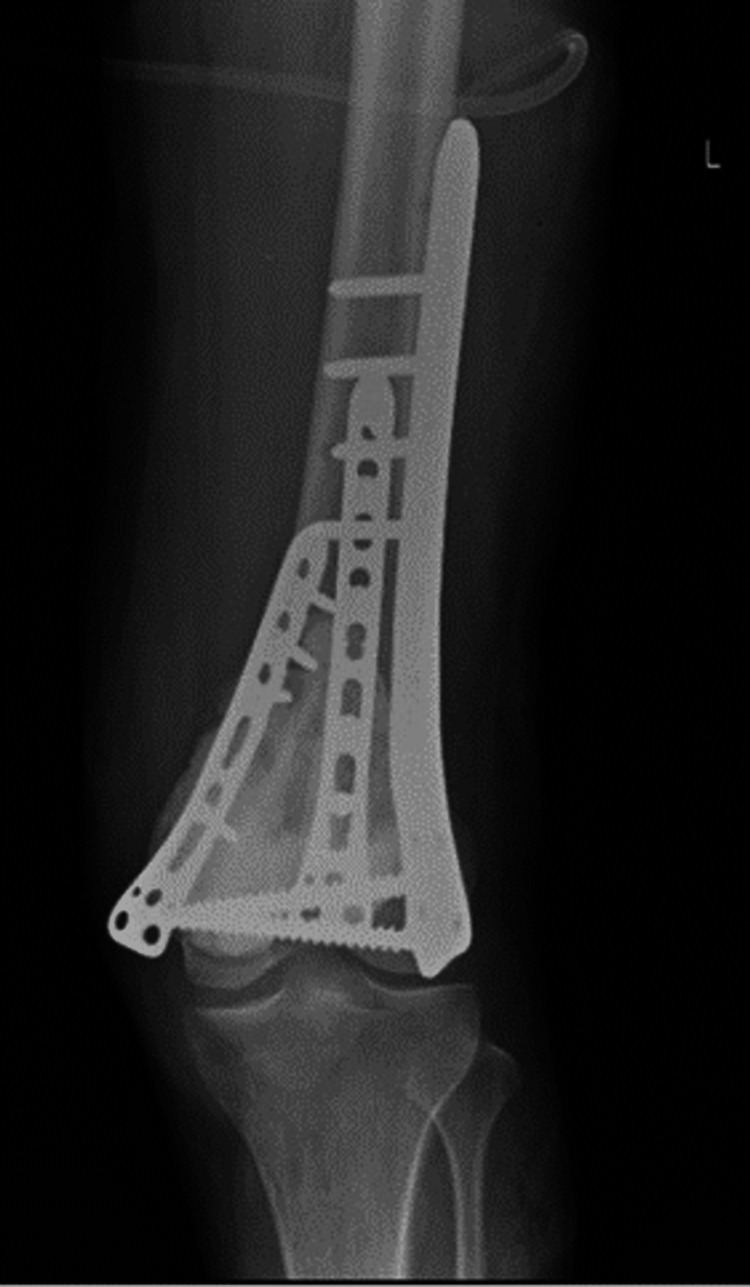
Postoperative plain radiograph, anteroposterior view of the left knee with 3-plate fixation

**Figure 8:**
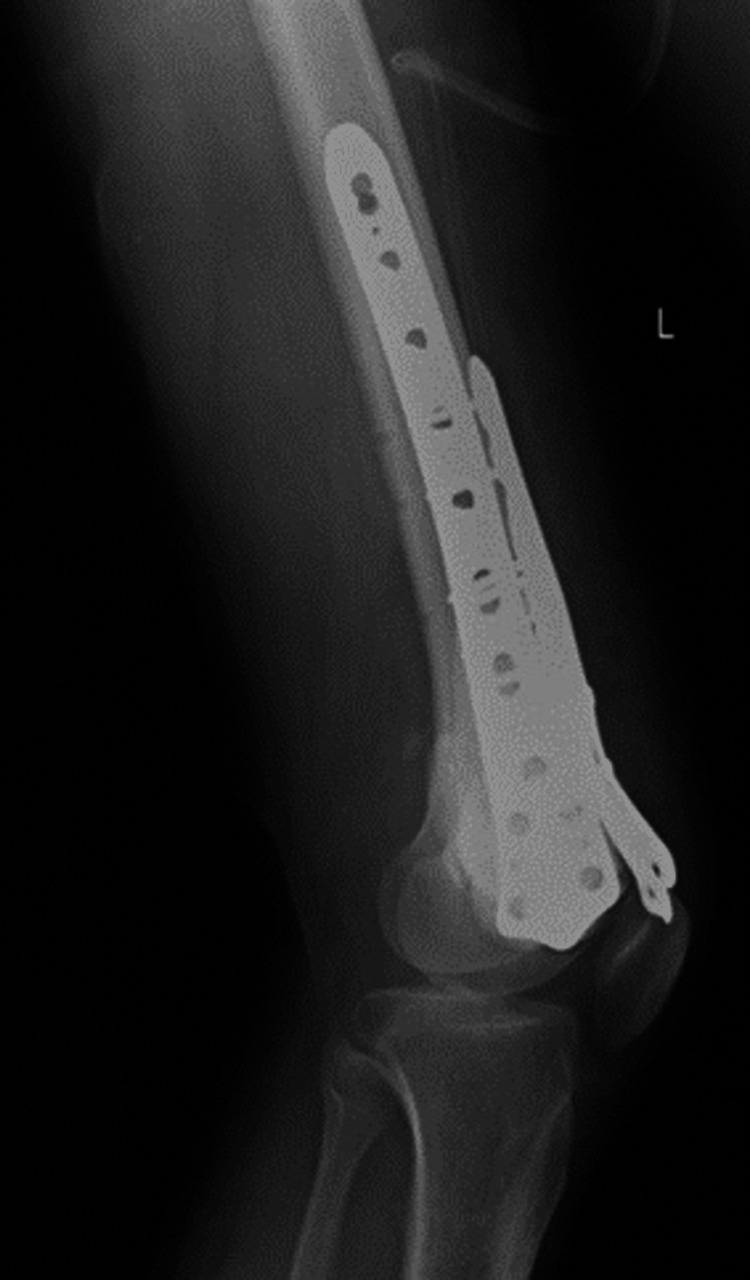
Postoperative plain radiograph, lateral view of the left knee with 3-plate fixation

**Figure 9:**
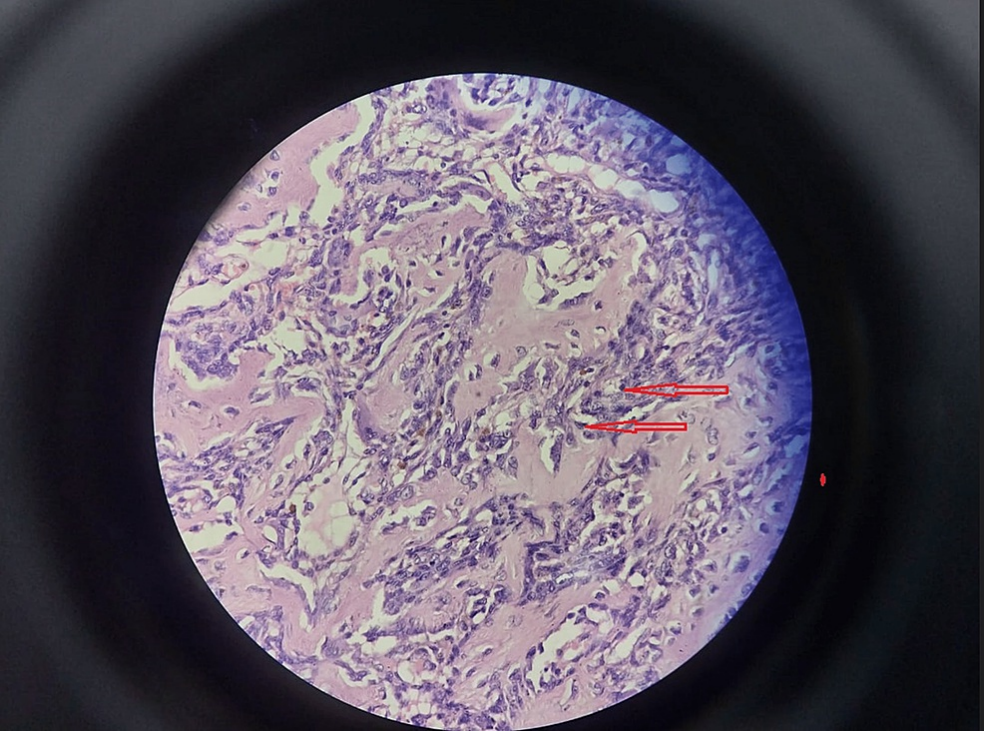
High-grade spindle-cell osteosarcoma composed of masses of fibrosarcoma-like, malignant spindle cells (H&E, x250)

Two weeks later, the patient was managed with curettage and internal fixation with plate osteosynthesis (Figures 7, 8). A bone graft filled the large bony defect from the iliac crest. The samples were sent for a histopathology examination. The report suggested osteogenic sarcoma or malignant transformation of GCTs. Sections from the tissue showed osteocartilagenous tissue with a tumor rim showing malignant spindle cells with large nuclei, nucleoli, and abnormal chromatin clumping along with giant cells, positive for infiltration by malignant cells on histopathology. There were also highly atypical mitoses, up to 5-10 per high power field (Figure 9). The patient was then scheduled for chemotherapy after 28 days of excision.

## Discussion

Although GCTs are frequently found, they rarely become malignant. The term "primary malignant giant cell tumor" refers to a malignant bone tumor with sections of typical benign cell tumor interspersed throughout and has never undergone curettage or radiation therapy. Usually, with a prior history of radiation therapy, curettage, or excision, the phrase secondary malignant GCT of bone is used to describe a malignant bone tumor that exhibits sarcomatous growth at a place where it was previously reported as a benign giant cell. The causes of subsequent malignant giant cell tumors of the bone that develop after radiation and curettage have been the subject of various theories [1,3,6]. Sakkers et al. proposed a theory that stated that post-curettage malignant transformation of GCT of bone was due to the proliferative changes at the margins of a dead bone which serves as a stimulus for the formation of a malignant tumor [2].

Brien et al. [1], in their report, state that there exist three different cell lines in a giant cell tumor of the bone. First, mononuclear spindle cells undergo proliferation in culture; second, non-spindle mononuclear cells do not proliferate in culture. Third, multinucleated giant cells are similar to osteoclasts and are formed due to the coalition of mononuclear stromal cells. The mononuclear cells and giant cells are derivatives of the hematopoietic system. They state that out of these three cell types, at least one is derived from the mesenchymal system, which is most likely to be the mononuclear spindle cells due to their ability to proliferate in culture [1,3]. A GCT is thus a mixture of cells that are derived from hematopoietic as well as mesenchymal origin. This explains the occasional conversion of a GCT into osteogenic sarcoma.

Various studies of malignant transformation of GCTs conducted previously have reported either a previous history of radiotherapy, curettage, or malignancy that occurred around 1.7 to 15 years after treatment of GCT [4,6,7]. Boriani et al. studied 327 GCTs and observed 10 cases of malignant transformation. Out of these, eight cases had a history of previous radiotherapy or curettage, and two cases transformed into sarcoma after many years of treatment [5,8,9]. A GCT that has been malignant from the beginning is extremely rare. In this case, the patient gave a history of complaints for only one year, which had got aggravated over the last month. The clinical findings, X-rays, and MRI studies suggested an aneurysmal bone cyst or secondary changes of aneurysmal bone cyst in a GCT. The biopsy specimen findings were consistent with these findings. However, after the complete excision of the tumor, a sarcomatous component was also interspersed with benign tumor cells. Thus, the tumor was malignant from the beginning, which is an infrequent occurrence. This can be explained by the possibility of the biopsy being taken from a site that was not representative of the tumor. This can happen due to the presence of areas of conventional GCT interspersed within osteosarcoma, which often makes the diagnosis difficult. Hence, the primary malignant giant cell bone tumor often mimics GCT clinically and radiographically.

## Conclusions

Though there is a possibility that conventional GCT will undergo malignant transformation, it is an extremely rare occurrence and it usually occurs after a history of radiation therapy, curettage, or excision. There is insufficient data in the literature to support the theory of spontaneous malignant transformation of GCTs of the bone. In our case, we arrive at the conclusion that the biopsy was taken from a site that was not representative of the osteosarcoma, and hence the diagnosis was missed and interpreted as a conventional GCT of the bone. Thus, osteosarcoma can mimic GCTs of the bone due to interspersed areas of conventional GCT within the tumor.
